# Assessment of an automated capillary system for *Plasmodium vivax* microsatellite genotyping

**DOI:** 10.1186/s12936-015-0842-9

**Published:** 2015-08-21

**Authors:** Paulo Manrique, Mari Hoshi, Manuel Fasabi, Oscar Nolasco, Pablo Yori, Martiza Calderón, Robert H. Gilman, Margaret N. Kosek, Joseph M. Vinetz, Dionicia Gamboa

**Affiliations:** Malaria Laboratory, Institute of Tropical Medicine Alexander von Humboldt, Universidad Peruana Cayetano Heredia, Lima, Peru; Division of Infectious Diseases, Department of Medicine, University of California San Diego, La Jolla, CA USA; Department of International Health, Johns Hopkins School of Public Health, Baltimore, MD USA; Instituto Nacional de Salud, Lima, Peru; Departamento de Ciencias Celulares y Moleculares, Universidad Peruana Cayetano Heredia, Lima, Peru

**Keywords:** Malaria, Molecular biology, Genotyping, Microsatellites, Molecular epidemiology, Technology

## Abstract

**Background:**

Several platforms have been used to generate the primary data for microsatellite analysis of malaria parasite genotypes. Each has relative advantages but share a limitation of being time- and cost-intensive. A commercially available automated capillary gel cartridge system was assessed in the microsatellite analysis of *Plasmodium vivax* diversity in the Peruvian Amazon.

**Methods:**

The reproducibility and accuracy of a commercially-available automated capillary system, QIAxcel, was assessed using a sequenced PCR product of 227 base pairs. This product was measured 42 times, then 27 *P. vivax* samples from Peruvian Amazon subjects were analyzed with this instrument using five informative microsatellites. Results from the QIAxcel system were compared with a Sanger-type sequencing machine, the ABI PRISM^®^ 3100 Genetic Analyzer.

**Results:**

Significant differences were seen between the sequenced amplicons and the results from the QIAxcel instrument. Different runs, plates and cartridges yielded significantly different results. Additionally, allele size decreased with each run by 0.045, or 1 bp, every three plates. QIAxcel and ABI PRISM systems differed in giving different values than those obtained by ABI PRISM, and too many (i.e. inaccurate) alleles per locus were also seen with the automated instrument.

**Conclusions:**

While *P. vivax* diversity could generally be estimated using an automated capillary gel cartridge system, the data demonstrate that this system is not sufficiently precise for reliably identifying parasite strains via microsatellite analysis. This conclusion reached after systematic analysis was due both to inadequate precision and poor reproducibility in measuring PCR product size.

**Electronic supplementary material:**

The online version of this article (doi:10.1186/s12936-015-0842-9) contains supplementary material, which is available to authorized users.

## Background

Molecular markers are widely used in the study of malaria and other infectious diseases [1]. Such markers allow for the differentiation of new and recurrent infections, tracing spread of clones of a pathogenic microorganism and discovering new introductions of infection on scales both large and small [2–5]. Molecular markers allow for genetic diversity studies of pathogen populations, their substructure and relationships among those sub-populations. Such data underpin fine scale analysis of infectious disease transmission dynamics [6–13], and allow us to infer the disease history and pathogen migration patterns, of particular importance in current malaria control- and elimination-focused research [14–17].

Different methods and markers have been used to study *Plasmodium* genetic diversity and population structure. A key feature of such studies is that various marker types evolve at different rates, being driven by complex mechanisms [10]. With regard to *Plasmodium vivax*, markers have included PCR–restriction fragment length polymorphism (PCR–RFLP) using a variety of genes including *PvCSP, PvMSP3*-*α* [18–20] and *PvMSP3*-*β* [8, 9, 19–23]. Another marker type is based on DNA sequence and single nucleotide polymorphisms (SNPs), which have been emphasized for global comparisons [15, 16, 24]. In recent years, parasite microsatellites markers have been used specifically to assess population diversity and structure [10–13, 25, 26], because large numbers of such markers are present in *Plasmodium falciparum* and *P. vivax* genomes [24, 27–29]. Microsatellites are presumed to be neutral if they are not physically linked to genes under selection [30–32], and have sufficiently high mutation rates to allow for the identification of epidemiologically relevant diversification events over relatively short periods of time [10].

While different platforms and techniques have been used to generate the raw data for microsatellite genotyping, they tend to be expensive. Costs include fluorophore-labelled primers and a capillary electrophoresis platform, typically a Sanger-type sequencing machine. In this study, the precision and reproducibility of the QIAxcel Advanced System from Qiagen [33] was evaluated using a well-characterized, gold standard sequenced PCR product followed by analysis of 271 *P. vivax*-infected blood samples from subjects living in the Peruvian Amazon. This platform was chosen based on reported performance characteristics primarily because fluorophore-labelled primers were not needed (hence the potential for cost saving). The main conclusion of this work, based on exhaustive analysis, should be a cautionary message for resource-limited settings, where reliance on hardware must be presaged by proper performance validation prior to investment of resources.

## Methods

### Ethical considerations

This study was approved by the Human Subjects Protection Program of the University of California, San Diego and the Comité de Ética of the Universidad Peruana Cayetano Heredia. Subjects provided written informed consent to participate in the study.

### *Plasmodium vivax* samples

**Fig. 1:**
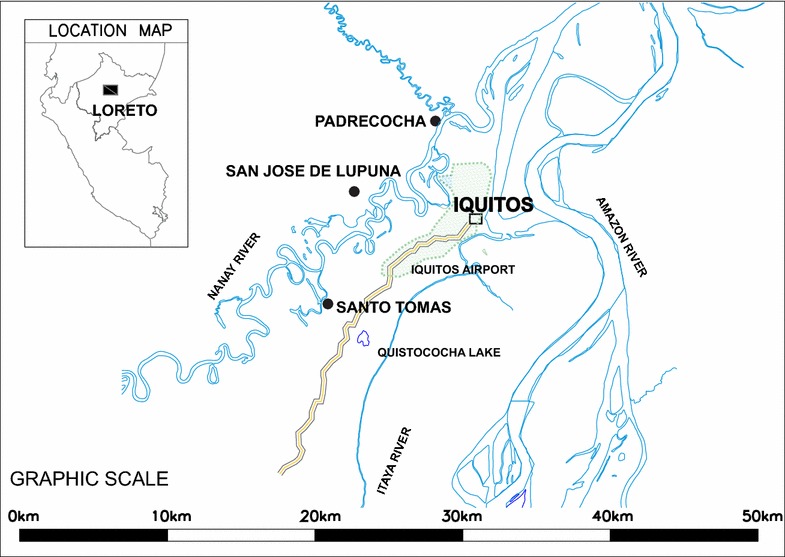
Map of the study sites

Samples for this study were obtained from three rural villages in the Peruvian Amazon region of Iquitos, Peru, in the Loreto Department, between 2005 and 2008: Santo Tomás (latitude −3.8094, longitude −73.3419,), San José de Lupuna (latitude −3.74611, longitude −73.3256) and Padrecocha (latitude −3.6997, longitude −73.2786). Rural communities are located 9.8, 9.9 and 21.1 km from Iquitos, respectively (main urban area in the region); San José de Lupuna and Padrecocha are accessible only by boat (Fig. 1).

Malaria diagnosis was carried out by microscopy using standard methods; for this reason only individuals with patent *P. vivax* parasitaemia were included in the study. Blood was obtained by venipuncture into EDTA tubes. Blood samples were frozen at −20 °C and shipped on dry ice to Universidad Peruana Cayetano Heredia, Lima, Peru for laboratory analysis. All individuals that tested positive were treated with primaquine (1 week, 15 mg/kg daily plus chloroquine) according to policies of the National Malaria Control Programme, Peruvian Ministry of Health.

### Accuracy, repeatability and reproducibility

One pGEM T-Easy plasmid containing a region of gene *Pf 18 S rRNA* was amplified by PCR using forward primer PL1473F18 [5′-TAA CgA ACg AgA TCT TAA-3′] and reverse primer PL1679R18 [5′-gTT CCT CTA AgA AgC TTT-3′] [34]. This PCR product was sequenced on the ABI PRISM 3100 Genetic Analyzer, then run 42 times in 42 different plates among four different cartridges on the QIAxcel Advanced System machine. In this way data obtained from sequencing and from the QIAxcel Advanced System machine were compared to assess reproducibility. Statistical analysis was done using STATA 12 (College Station, TX, USA). Student’s T test was used to test differences among outputs from different runs, plates and cartridges used on the QIAxcel platform, and to test differences between the QIAxcel with the reference gold standard method ABI PRISM 3100 Genetic Analyzer. To measure the effect of cartridge use on the outputs, a generalized linear mixed regression analysis was made [35]. To test whether the variance of the results from the QIAxcel machine was greater than expected compared to the ABI PRISM System, a one sample test of variance was used [36], assuming a maximum value of the standard deviation of the ABI PRISM system of 0.5 base pairs; additionally, the test was also run assuming a maximum standard deviation of one base pair. Finally, the accuracy of this QIAxcel platform was measured using five different microsatellite targets on 27 *P. vivax* samples. Three microsatellites (MS4, MS9 and MS15) were non-degenerate or perfect, indicating a unique motif repeat. The other two microsatellites were imperfect with mixed motifs; combination between discontinuous motifs, that are trunked with non-repetitive regions, and compounds motifs, with two or more different motifs. Each PCR product was analyzed in both platforms the QIAxcel Advanced System machine and the ABI PRISM system, and three replicates of MS9 and MS15 were done. Results differences between platforms were tested using Student’s T test in STATA 12.

### Microsatellite genotyping

Genomic DNA was isolated from whole blood using the QIAamp DNA Blood Kit (QIAGEN, Valencia, CA, USA). Samples were genotyped using a set of 15 previously reported microsatellite markers [37, 38]. Five microsatellites were used to genotype 27 samples in a test for accuracy (MS4, MS6, MS9, MS15 and MS20), and all microsatellites were used to measure genetic diversity using 271 *P. vivax* samples (primers are listed in Additional file 1). Five microliters of template DNA was used for PCR in a 50 μl total volume for each individual reaction. The PCR product was then separated on a QIAxcel Advanced System (QIAGEN). The QIAxcel Advanced System from Qiagen is commercially presented as a novel means to replace gel analysis of DNA, RNA, and proteins. It consists of 12 independent capillaries placed in a cartridge. Each cartridge allows processing about 100 samples per capillary and 96 samples per PCR plate. This system is marketed to have a resolution of 3–5 bp. The PCR product sizes were estimated using the QIAxcel ScreenGel Software.

### Capillary electrophoresis analysis

Electrophoresis runs on the QIAxcel Advanced System (QIAGEN) were performed according to manufacturer’s recommendations. For each electrophoresis process, a QIAxcel DNA High Resolution Cartridge (Cat. no. 929002), a QX Alignment Marker 15 bp/600 bp (Cat. no. 929530), a standard QX DNA size marker 25–500 pb v2.0 (Cat. no. 929560), and the OM 700 method, were used. Each cartridge was calibrated using QX Intensity Calibration Marker (cat. no. 929500) for its first use. Because five plates were processed per day (40 runs), the Separation buffer, the Wash buffer and the Alignment Marker on the buffer tray were renewed every day. The first well (position A1) of each plate was filled with 20 μl of one-twentieth dilution of the standard QX DNA Size Marker 25–500 bp. The second well (position A2) was filled with Pf 18s rRNA PCR product as a reference control. The estimated size of this product was used to confirm or correct the size of the samples between plates. The third and fourth well was filled with the positive and negative PCR control samples, respectively. The positive control was a monoclonal sample with high parasitaemia used to confirm the effectiveness of the PCR. It was also used to see the presence of non-specific and stutters products. Additionally, this product also helped to correct the inferred size of the samples.

ABI PRISM system electrophoresis runs were carried out using a 4-capillary array of 36 cm in combination with 3130 POP-4TM Polymer. The GeneScan 500 LIZ dye was used as Size Standard, and primers were labelled with 6FAM, VIC, NED and PET dyes. Each plate well was filled with 0.25 μl of GeneScan 500 Liz, 9.25 μl of Hi-Di Formamide, and 1 μl of one-tenth dilution of the PCR product. A monoclonal sample was used as positive control, and Human DNA as negative control.

### Microsatellite analysis

The PCR product sizes were estimated using the QIAxcel ScreenGel Software and the GeneMapper v3.0 for QIAxcel and ABI PRISM results, respectively. Peaks with more than 50 RFU (Relative fluorescence units) in the electropherogram were interpreted as alleles, and if additional peak of at least one-third the height of the predominant peak was seen it was also scored as allele. Finding more than one allele was interpreted as a polyclonal infection containing two or more genetically distinct clones in the same sample. Missing data (no amplifications) was reported by loci but were not considered here for defining haplotypes.

Microsatellite data were entered and cleaned using Microsoft Excel, then formatted using an Excel complement called GenAlEx 6.501 [39]. The expected heterozygosity (He) parameter was estimated, defined as:

$${\text{H}}_{\text{e}} = \left[ {{\text{n}}/\left( {{\text{n}} - 1} \right)} \right]\left[ {1 - \mathop \sum \limits_{{{\text{i}} = 1}}^{\text{L}} {\text{p}}_{\text{i}}^{2} } \right],$$

where n is the number of isolates analyzed and pi is the frequency of the ith allele (i = 1,…, L) in the population [40].

Expected heterozygosity (He) gives the average probability that a pair of alleles randomly selected from the population is different. Haplotypes, described as unique allelic combinations of the 15 loci analyzed, were determined in monoclonal samples using GenAlEx.

## Results

### Accuracy, repeatability and reproducibility of the QIAxcel Advanced Capillary System

**Fig. 2:**
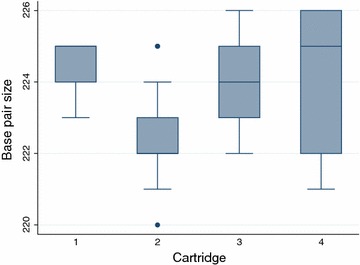
Distribution of the results of the sequenced PCR product, run 42 times in four different cartridges

A sequenced PCR product of 227 base pairs (Additional file 2) was run 42 times in four different cartridges (5, 13, 13, and 11 times per each cartridge) on the QIAxcel Advanced System, giving a mean value of 223.619 base pair (SD = 1.62229), and a range from 220 to 226 bp. There were significant differences between the sequenced values and the results from the QIAxcel Advanced System (Student’s t test, *p* = 0.0029). Moreover, there were significant differences between different runs, plates and cartridges (Student’s t test, *p* = 0.0275) (Fig. 2). Additionally, 18 samples were run at least five times in the same run to test reproducibility among capillaries. Using the ‘one sample test of variance’ showed significant differences even at a maximum standard deviation value of 1 (*p* = 0.0003).

**Fig. 3:**
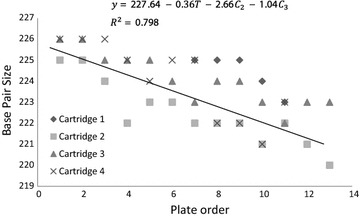
Effect of the time of use on the outcome obtained from the QIAxcel Advanced System. Y-axis is the result of the QIAxcel (the results of the sequenced product) with four different cartridges, and the X-axis is the time order of each plate which was ran in the QIAxcel. T is the time order of the plate in each cartridge and −0.36 is its effect on the outcome. C2, C3 and C4 are the second, third and fourth used cartridges and the values represent their effect on the outcome in respect to the first cartridge

The effect of the time of use of each cartridge was explored by using a mixed linear regression analysis. A statistically significant decrease of -0.0449 base pairs from one run to the next (95 % CI −0.0533 to −0.0365) was observed in the allele size each time a sample was evaluated; and a decrease of −0.3595 base pairs (95 % CI −0.4267 to −0.2922) was found each time a plate was run. Furthermore, from the four cartridges used, the results given by the second cartridge were −2.657 base pairs (95 % CI −3.447 to −1.868) lower than those given by the first one (Fig. 3). In the case of the third and fourth cartridges the results were −1.042 (95 % CI −1.832 to −0.252) and −1.387 (95 % CI −2.21 to −0.565) lower, respectively.

Comparing analysis of five microsatellites (MS4, MS6, MS9, M15 and MS20) from 27 samples, the automated QIAxcel advanced system showed higher size values than those obtained by the ABI PRISM system. On average, QIAxcel’s size values were 7.022 base pairs higher (95 % CI 6.34–7.7). To test if differences between platforms were due to the label of primers, MS9 and MS15 were analyzed using both kind of primers in triplicates, and no significant differences were seen in MS9 products; however, significant differences were seen in MS15 products (p value <0.0001). The numbers of alleles for MS4, MS6, MS9, MS15 and MS20 on the ABI PRISM system were 3, 2, 4, 3 and 4, respectively. The corresponding allele numbers on the QIAxcel system were 7, 6, 5, 4 and 8, respectively (summarized in Table 1; complete results in Additional file 3). Three of the 27 samples were polyclonal by ABI PRISM, but only two were detected as such by the QIAxcel system. Analysis of microsatellites MS6 and MS20 detected all polyclonal samples using the ABI PRISM system, while MS15 was not. The other two markers did not detect any polyclonal sample. On the QIAxcel system only microsatellite MS15 was able to detect the polyclonal samples.

**Table 1:** Comparison of results using QIAxcel vs. ABI PRISM methods

Allele	MS4		MS6		MS9		MS15		MS20	
	ABI	QIAxcel	ABI	QIAxcel	ABI	QIAxcel	ABI	QIAxcel	ABI	QIAxcel
1	187 (0.19)	190 (0.08)	210 (0.63)	219 (0.39)	147 (0.19)	156 (0.19)	243 (0.69)	247 (0.24)	200 (0.1)	209 (0.04)
2	193 (0.63)	191 (0.08)	229 (0.33)	220 (0.18)	155 (0.04)	165 (0.11)	249 (0.14)	248 (0.45)	206 (0.2)	210 (0.07)
3	200 (0.19)	192 (0.04)		221 (0.07)	157 (0.11)	166 (0.04)	258 (0.17)	254 (0.14)	220 (0.07)	211 (0.04)
4		197 (0.32)		233 (0.04)	163 (0.67)	172 (0.60)		262 (0.17)	222 (0.63)	216 (0.15)
5		198 (0.36)		237 (0.14)		173 (0.07)				218 (0.04)
6		203 (0.08)		238 (0.18)						221 (0.04)
7		204 (0.04)								230 (0.44)
8										231 (0.19)

The table shows the base pair size of each allele. In parentheses the frequencies of each allele are denoted

### Analysis of *Plasmodium vivax* samples from the Peruvian Amazon

A total of 271 *P. vivax* single infection samples were analyzed using samples obtained from three rural communities around Iquitos (Padrecocha, San José de Lupuna and Santo Tomás) from the years 2005 and 2008. In Padrecocha, 50 blood samples were taken in the year 2005; 50 blood samples were available from 2006. In San José de Lupuna, 15 blood samples obtained in 2006; 35 blood samples were from 2007; 20 blood samples were from 2008. From Santo Tomás, 40 blood samples were obtained in 2006; 30 blood samples in 2007; and 31 blood samples in 2008 (Table 2).

**Table 2:** Number of monoclonal and polyclonal samples by study site

Locality	Year	Monoclonal	Polyclonal	Total
Padrecocha	2005	43 (86 %)	7 (14 %)	50
	2006	44 (88 %)	6 (12 %)	50
	Total	87 (87 %)	13 (13 %)	100
San Jose de Lupuna	2006	14 (93 %)	1 (7 %)	15
	2007	26 (74 %)	9 (26 %)	35
	2008	16 (80 %)	4 (20 %)	20
	Total	56 (80 %)	14 (20 %)	70
Santo Tomas	2006	30 (75 %)	10 (25 %)	40
	2007	26 (87 %)	4 (13 %)	30
	2008	23 (74 %)	8 (26 %)	31
	Total	79 (78 %)	22 (22 %)	101
Total		222 (82 %)	49 (18 %)	271

### Estimation of *Plasmodium vivax* genetic diversity

Two-hundred and seventy-one *P. vivax* samples were genotyped using the automated QIAxcel Advanced System machine. Of the 271 *P. vivax* samples, 49 samples were polyclonal; 13 (15 %) polyclonal infections were found in Padrecocha, 14 (25 %) in San José de Lupuna and 22 (28 %) in Santo Tomás (Table 2). Moreover, of the 222 monoclonal samples, 141 amplified all loci, 53 showed one null allele, 22 showed two null alleles, five showed three null alleles, and just one sample did not amplify 14 loci out of the 15 loci analyzed (Table 3).

**Table 3:** Number of missing data in each study site

Number of missing data	Padrecocha		San Jose de Lupuna		Santo Tomas		Total		
	Null alleles	Undefined alleles	Null alleles	Undefined alleles	Null alleles	Undefined alleles	Null alleles	Undefined alleles	Missing data (%)
0	54	47	48	32	39	40	141	119	72 (32.4)
1	23	22	6	18	24	29	53	69	72 (32.4)
2	7	9	2	6	13	7	22	22	49 (22.1)
3	3	6	0	0	2	1	5	7	16 (7.2)
4	0	1	0	0	0	2	0	3	9 (4.1)
5	0	2	0	0	0	0	0	2	3 (1.4)
14	0	0	0	0	1	0	1	0	1 (0.5)
Total	87	87	56	56	79	79	222	222	222 (100)

*Null allele*: no PCR amplification of a sample.* Undefined allele*: PCR product not assigned to an allele. *Missing data*: allele classified as Null allele or Undefined allele

**Fig. 4:**
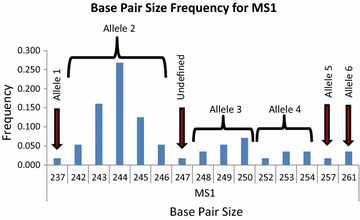
Allele assignments. Displayed are the frequencies of the different products obtained for microsatellite MS1 by the QIAxcel Advanced System, and its assignment to alleles. The product 237 was defined as allele 1 since it was separated by five bases from the product 242. The products from 242 to 246 were defined as allele 2 because these products were consecutive and the frequency rise from 242 to 244, and then it decrease at 246. The products from 248 to 250 were defined as allele 3; and the product 247 was labelled as undefined since it could be assigned to allele 2 as well as to allele 3

Because of the insufficient accuracy and reproducibility obtained using the QIAxcel Advanced System, the base pair size result was transformed into an allele profile and coded. In this way, all base pair size results were ranked by size and a frequency histogram was made per each locus. Base pair sizes with consecutive results (for example, 242, 243, 244, 245, 246 bp) were grouped into one unique allele based on frequency (Fig. 4). There were some base pair size results that were in the middle of two alleles and hence could not be defined. Each base pair size result that was not defined was labelled as zero or a null allele. As a result, of the 222 monoclonal samples, 103 samples had at least one undefined allele and just 72 samples had a complete profile (Table 3). Most microsatellites except MS4, MS5, MS15, and PV6635 lacked at least 5 % of possible data (MS16 presented the highest missing data value with 18.55 %).

The He index of samples from each geographical source of sample was 0.744 ± 0.039, 0.734 ± 0.056 and 0.716 ± 0.052, for Padrecocha, San José de Lupuna and Santo Tomas, respectively; He for the whole population was 0.779 ± 0.04. Microsatellite MS7 showed the lowest diversity in the whole population (He = 0.375) as well as in the three different locations (He for MS7 in Padrecocha, San José de Lupuna and Santo Tomás was 0.536, 0.170, and 0.296, respectively). MS5 showed low diversity (He = 0.539, 0.343 and 0.399, respectively); however, its diversity was higher among the whole population (He = 0.621). Microsatellites MS3 and MS1 showed a similar patterns. Microsatellites MS16, MS9 and MS8 showed the highest genetic diversity among localities and in the whole population (average He = 0.92, 0.91 and 0.902, respectively). Moreover, many of the alleles from the different markers used were in low frequency (frequency lower than 5 %, referred as minor allele frequency or MAF, Table 4).

**Table 4:** Number of alleles and genetic diversity by locus by site

Pop	Padrecocha				San Jose de Lupuna				Santo Tomas			
Locus	N	Na	MAF (%)	He	N	Na	MAF (%)	He	N	Na	MAF (%)	He
MS1	70	5	2 (40)	0.659	55	6	4 (67)	0.52	63	6	4 (67)	0.467
MS2	77	7	4 (57)	0.651	56	8	3 (38)	0.812	64	8	3 (38)	0.8
MS3	84	2	0 (0)	0.413	53	6	2 (33)	0.761	72	4	2 (50)	0.476
MS4	86	9	4 (44)	0.826	55	6	3 (50)	0.71	77	9	4 (44)	0.822
MS5	87	4	2 (50)	0.539	56	2	0 (0)	0.341	78	2	0 (0)	0.399
MS6	74	7	2 (29)	0.811	53	6	1 (17)	0.816	77	6	2 (33)	0.766
MS7	77	5	3 (60)	0.536	56	4	3 (75)	0.17	66	3	2 (67)	0.296
MS8	74	11	4 (36)	0.873	49	12	6 (50)	0.854	76	13	7 (54)	0.841
MS9	72	11	3 (27)	0.894	53	9	2 (22)	0.864	73	12	5 (42)	0.866
MS10	85	12	8 (67)	0.734	49	11	5 (45)	0.838	58	13	8 (62)	0.817
MS12	81	11	7 (64)	0.779	52	13	4 (31)	0.912	76	10	4 (40)	0.838
MS15	84	8	3 (38)	0.81	55	7	3 (43)	0.789	75	7	3 (43)	0.739
MS16	70	18	9 (50)	0.902	48	15	7 (47)	0.895	62	15	6 (40)	0.901
MS20	82	8	2 (25)	0.844	55	12	3 (25)	0.905	68	13	4 (31)	0.9
Pv6635	84	12	4 (33)	0.891	54	9	4 (44)	0.82	73	9	4 (44)	0.814

*N* number of samples that amplified per locus, *Na* number of alleles, *MAF* number of alleles at minor allele frequency (frequency less than 5 %), *He* unbiased expected heterozygosity

Among the 72 monoclonal infections with complete profiles, 62 haplotypes were found, of which 53 had unique haplotypes. None of the haplotypes were shared among the three populations, but one haplotype was shared in San José de Lupuna between the years of 2007 and 2008.

## Discussion

This study aimed to explore the utility of a non-fluorophore-dependent, automated microsatellite analysis system that purportedly had two major advantages—higher throughput and lower cost—to obtain raw microsatellite marker data. However, the use of microsatellite data, like that of other markers [22], requires high precision and reproducibility. Hence, any platform used to generate primary molecular marker data must be able to differentiate alleles of close but non-identical size, such as those in the present study that differ in size by 3 bp or less. The QIAxcel Advanced System did not demonstrate sufficient reproducibility or accuracy (i.e., to measure the bp of a sequenced PCR product (range of ±3 base pairs)) to enable accurate data interpretation. These results were largely similar to previous (but smaller scale) studies [41]. The data provided here was based on comparison of a gold standard—a sequenced 227 bp PCR product—and using a sequencing gel measurement as a benchmark. This PCR product did not show any stutter or non-specific product, errors which are common in microsatellite analysis. Therefore, with microsatellite products with genotyping errors (i.e., stutters, additionally added adenines, non-specific peaks) the range of variation could be higher as the presence of stutters could affect the migration patterns of molecules on the capillary electrophoresis. In this sense, most of the degenerated microsatellite markers used in this study showed a great number of non-specific products, especially MS8 which showed four to six non-specific products near the principal allele peak.

Discrepancies between ABI PRISM and QIAxcel results were also seen. Twenty seven samples were genotyped by both platforms using five microsatellites markers. QIAxcel not only gave different product sizes than those observed on the ABI PRISM, but more, inaccurate alleles per locus were produced, which altered potential data interpretation (Additional file 3). Even though results were different, they were correlated between each other and those additional alleles observed were easily detected and edited to its correct allele size. However, when more replicates were done the variation of results increase, making more difficult to assign the correct value (Additional file 4). Differences between labelled and non-labelled products were seen too, but it was no as high as differences between QIAxcel and ABI platform (Additional files 5, 6). This heterogeneity of results was reflected in the analysis of the 271 samples. This analysis resulted in high diversity of markers and high number of alleles per locus. Though most markers were supposed to differ in three base pairs, differences in just one base pair were usually seen. The error produced by the QIAxcel Advanced System was reduced by transforming raw data to allele profile codes. Even though the expected He in each locality was lower and probably more accurate using the transformation (i.e., more lumping, less splitting), the genetic diversity of the three localities found in this study was somewhat higher than previously reported in the same region using the same markers but standard methods based on fluorophore-labelled primers [11, 25, 42].

Another limitation to the generalizability of the results presented here is the high rate of missing data that was found in 13 microsatellite markers. A particular case was MS16, which showed that 18 % of data points were missing (72 % due to null alleles), higher than for the other microsatellites. This result possibly suggests that a mutation in the annealing region of one of these primers did not allow this product to amplify. Because of the high rate of missing data (more than 5 % for 13 of the 15 microsatellites used) and the overestimation of diversity, more in-depth analyses, such as pair-wise linkage disequilibrium and genetic differentiation between populations were not able to be performed since those could lead to erroneous interpretations.

Current malaria epidemiology research relies substantially on the use of molecular markers to estimate the malaria transmission dynamics. For this reason, genotyping methods with low cost of reagents, high accuracy and repeatability are needed. Recently analysis of single nucleotide polymorphisms (SNPs) have been shown to reliably to infer malaria transmission patterns in hyper-endemic regions [43]. However, in low transmission regions where the parasite diversity is low associated with a clonal expansion of parasites, microsatellites are still the primary tool for detecting the small differences between parasite strains so that one can correctly infer transmission dynamics and potential outcomes of control measures [7, 10, 11, 29].

The automated QIAxcel advanced system is marketed as a rapid, facile and low-cost method for genotyping. Its main advantage is that it not depends on labelled primers with fluorophores which reduce the cost of the genotyping process drastically. While a 100 nano moles of a common primer is around 10 dollars, just 10 nano moles of a labelled primer is around 80 dollars. This difference makes the QIAxcel System an attractive option, but due to this characteristic, it is not possible to run the size marker and the sample into the same well and just one well contain the size marker in a plate, and the other wells contain the samples and the alignment markers. This work shows that each independent capillary and cartridge has different patterns of migration, and that the variation of results increases over the time. This heterogeneity is supposed to be reduced with the use of an alignment marker that normalizes the pattern of migration of molecules between capillaries, cartridges and runs. As the alignment marker flanks the PCR product in a range of 600 nucleotides the normalization process is not accurate enough to differentiate products that differ in 3 bp.

## Conclusion

The use of affordable platforms that can generate data from fast evolving molecular markers, such as microsatellites, could facilitate scaling up molecular epidemiologic investigations in *P. vivax* and other eukaryotic pathogens. Several platforms and techniques have been used for this purpose; however, systematic assessment of the performance (precision, accuracy and reproducibility) of such systems is often lacking. This study found that one such system, the QIAxcel Advanced System, was not a reliable platform for *P. vivax* genotyping using microsatellites.

## Additional files

Additional file 1: Microsatellites analyzed: chromosomal location, motif, and primers. Additional file 2: A sequenced PCR product of 227 base pairs used. Additional file 3: Comparison of allele sizes using QIAxcel *vs* ABI Instruments. Additional file 4: QIAxcel analysis of microsatellites 9 and 15 revealed too many alleles due to imprecision. Additional file 5: Differences between labelled and unlabelled primers of Microsatellite 9. Additional file 6: Differences between labelled and unlabelled primers of Microsatellite 15.
