# Supplementary material for: Assessment of an automated capillary system for Plasmodium vivax microsatellite genotyping
Source: Malar J. 2015 Aug 21;14:326. doi: 10.1186/s12936-015-0842-9 (PMC4546211; doi:10.1186/s12936-015-0842-9)
Supplement: Additional file 2: — A sequenced PCR product of 227 base pairs used. [file 12936_2015_842_MOESM2_ESM.pdf]

TTAACGAACGAGATCTTAACCTGCTAATTAGCGGTAAGT
ACACTATATTTTTATTTGAAATTGAATATAGGTAATTATAC
ATGTTTATTCAGTGTTCAAATTAGGATATTTTTTTATTAAA
ATATTCTTTTCCCTGTTCTACTATAATAATTTGTTTTTTTTT
ACTCTATTTCTCTCTTCTTTTAAGAATGTACTTGTTTGATT
AAATAAAGCTTCTTAGAGGAACT
